# Reclaiming informed consent to train mental health AI with patient data

**DOI:** 10.1038/s41746-026-02843-8

**Published:** 2026-06-02

**Authors:** Carlos A. Larrauri, Nicole Martinez-Martin, John Torous

**Affiliations:** 1 Executive and Continuing Education, Harvard T.H. Chan School of Public Health, Boston, MA USA; 2 Department of Pediatrics, Stanford Center for Biomedical Ethics, Stanford, CA USA; 3 Digital Psychiatry Division, Beth Israel Deaconess Medical Center, Boston, MA USA

**Keywords:** Complex networks, Complex networks, Health care, Science, technology and society, Scientific community, Social sciences

## Abstract

Companies increasingly train AI models on therapy transcripts, often relying on terms of service that obscure data use to obtain assent. Because AI systems generate inferences from data and enable unforeseen secondary uses, existing consent models do not adequately protect autonomy and confidentiality. We argue for (1) separate and explicit opt-in consent to train on patient data and (2) patient-led governance to prevent the recurrence of exploitation in medical innovation.

When researchers obtained Henrietta Lacks’ cells without her consent in 1951, they transformed medicine while also creating a paradigmatic example of biomedical progress built on exploitation^1^. Today, we risk repeating a similar mistake, not with tissue, but with patients’ digital data. Companies developing artificial intelligence (AI) for mental health are using digital therapy transcripts of conversations with both human and AI therapists to train their models, often relying on lengthy terms of service to obtain assent. For instance, recent reporting from *STAT News* suggested that Lyra Health uses deidentified therapy transcripts to develop its new AI chatbot, which will be marketed to employers and health systems^2^. Lyra is not alone; other digital mental health platforms, including Headspace Health, Spring Health, and Talkspace, are similarly integrating generative AI into their services^3^. Details regarding the sources of their training data remain unclear.

Just as HeLa cells became indefinitely reproducible, generating scientific value across contexts in ways Henrietta Lacks or researchers could never have anticipated, patient data achieves a similar “immortality” once incorporated into AI systems, yet with even greater reach. Unlike a discrete tissue sample, patient data can be combined with other datasets to generate new inferences and create reidentification risks (linking data back to specific individuals), making downstream uses difficult to foresee or control. Henrietta Lacks’ story ultimately catalyzed significant reforms in research ethics and patient rights, and the current moment demands a comparable reckoning with the human costs of innovation, given that millions of people have likely already lost control over their most personal data. Reclaiming informed consent, therefore, requires an AI governance framework that ensures separate and explicit opt-in consent and patient-led governance to prevent the recurrence of historical patterns of exploitation in medical innovation.

## Why contractual assent is not informed consent

The therapeutic relationship is traditionally governed by professional ethics and legal duties^4^ that protect patient autonomy and confidentiality. Most patients in brick-and-mortar settings would not expect their private therapy sessions to be recorded; however, audio or video recordings of individual therapy sessions are occasionally made for the training or evaluation of human therapists, usually less than one session per year. Such recording requires explicit, direct, and prospective informed consent, with additional consent forms specifically for that purpose, and participants may rescind their consent at any time^5^. Unlike traditional therapy, digital mental health platforms can—and often do—record all sessions to train and improve their AI models, under ethical and legal safeguards far weaker than those governing conventional therapeutic settings. For instance, on January 6, 2026, the FDA issued guidance documents on low-risk devices^6^ and clinical decision support software^7^, suggesting that it will consider classifying more AI applications as “wellness” devices or software and thus outside the scope of formal regulation.

Understanding what this means for patients requires distinguishing among three frameworks: (1) informed consent for clinical research, governed by principles emphasizing disclosure, voluntariness, and comprehension^8^; (2) authorization regimes for secondary use of data, which dispense with individual consent by repurposing data beyond its original purpose through de-identification or institutional review board waivers in the United States^9^, or other legal grounds in Europe^10^; and (3) contractual assent embedded in terms of service. While informed consent in clinical research is the most rigorous of these frameworks, it only documents that information was disclosed, not that it was necessarily understood. Secondary health data sharing outside of research contexts typically operates under far less stringent requirements. Digital mental health platforms largely rely on the third and weakest model, which requires users to click “I agree” to terms that few read or understand^11^, and often reduces complex data permissions into either a blank check or refusal^12^. Those relying on employer-sponsored platforms such as Lyra Health face an even starker choice: accept the terms or forgo care.

But by opting in to get help, what is being forfeited? Informed consent requires information about both benefits and risks, yet AI complicates this calculus because the use and implications of the data collected can extend far beyond the immediate encounter^13^. Large language models (the AI systems underlying these modern chatbots) enable secondary uses of data, making it difficult to anticipate how information may later be applied^14^. Moreover, the inferences drawn from the data and the user and group profiles that can be derived from those inferences pose additional risks to individuals and groups, even when the underlying data is not directly identifiable^15^. For instance, employers or insurers could use collected data and resulting profiles to identify debilitating or costly conditions, thereby facilitating discrimination in employment or coverage decisions that patients did not anticipate when seeking care^16^. This risk may be further heightened where companies developing AI chatbots are contracted directly by the very employers whose workforces they serve.

## Chatbot therapy faces regulatory gaps and conflicted incentives

AI holds significant promise for improving psychiatric care, including enhancing access, augmenting clinical decision-making, and improving early risk detection^17^. Yet, current legal regimes inadequately protect patient data. In the United States, the Health Insurance Portability and Accountability Act (HIPAA) protects personal health information (PHI) collected in clinical encounters^18^, but many digital mental health platforms do not qualify as “covered entities” under HIPAA. As a result, regulators and private litigants must rely on a patchwork of authorities to address misleading or undisclosed data practices and privacy breaches, including Section 5 of the Federal Trade Commission Act, the Health Breach Notification Rule under the Health Information Technology for Economic and Clinical Health Act, state consumer protection and privacy laws, and federal and state wiretapping statutes. While the European Union’s General Data Protection Regulation (GDPR) provides broader baseline protections for personal data than the health sector-specific scope of HIPAA^19^, the EU has moved toward policies that harmonize secondary uses of health data without explicit consent. The GDPR already authorizes such processing for certain public interest, statistical, and scientific or historical research purposes, but member states implement these exceptions inconsistently. The European Health Data Space—an EU regulatory framework governing the cross-border sharing and reuse of personal health data—was initially proposed to permit standardized data sharing without consent, and only through late-stage advocacy were opt-out provisions secured^20^.

The question of deidentification is central to this debate, yet current regimes treat it as a bright-line rule that eliminates privacy concerns. Once data is deidentified under HIPAA’s standards, it is no longer legally considered PHI^21^. The GDPR’s “right to erasure” offers stronger data protections in principle, extending to pseudonymized and inferred data if linked to an individual (but not de-identified data). However, commentators note that it loses practical force once personal data has been integrated into complex, periodically updated systems, where erasure may be technically ineffective and inferred data or trained models persist despite withdrawal of consent^22^. Moreover, deidentification is not necessarily permanent: reidentification risk increases as public and commercial datasets proliferate and become linked to other sources^23^. Given these regulatory shortcomings, companies are best positioned to prevent harm, yet market dynamics that reward rapid scaling and data accumulation also create structural incentives to collect user data^24^, thereby risking the repetition of historical patterns in which marginalized populations contributed to medical progress without sharing its benefits or protections^25^.

## Reclaiming informed consent with separate and explicit opt-in consent and patient-led governance

Digital mental health platforms do not deviate radically from prevailing data practices; however, patient data in the mental health context is often generated during episodes of vulnerability and distress and is highly stigmatized. These features justify heightened consent standards, even if similar practices in other sectors remain subject to debate. Moreover, AI enables the collection and processing of far more data than people are aware of, a trend likely to continue as newer models integrate facial expressions, wearables, fitness data, voice sampling, and other potential mental health biomarkers. Informed consent must therefore transition from a procedural checkbox to an ongoing process, anchored in accountability and transparency, giving users genuine control over their data. Dynamic consent achieves this shift through a digital interface that enables participants to modify consent preferences over time while maintaining communication about data use and outcomes^26^. Related consent models, including meta consent—where individuals choose their preferred consent method for future data uses—offer an additional model for balancing autonomy with practical feasibility^27^.

We propose two complementary policies: one procedural and one structural. First, entities with authority to shape digital mental health practices, such as state and federal regulators and enforcement agencies, payers, and health systems, should mandate separate and explicit opt-in consent for the use of patient data in AI model training, clearly distinguished from consent to receive treatment. This consent documentation must explain, in accessible language, what data companies will use and derive, for what developmental purposes (e.g., developing a new chatbot), and through what commercial arrangements. While companies and health systems often argue that routine clinical care occurs under general consent—a broad, blanket authorization for treatment and associated operational data uses—and that consent for downstream data uses is infeasible and would hamper innovation, patient data derived from therapy transcripts is distinct from routine clinical care.

Second, communities whose data fuel these systems should be included in efforts to define their ethical boundaries and consent standards. AI governance models must redistribute authority and accountability to ensure sustained trust and legitimacy, with leadership roles establishing patient and community oversight. Patient advocacy organizations, such as the National Alliance on Mental Illness (NAMI), are well positioned to assist in embedding patient representation on governance boards^28^, algorithmic audit panels^29^, and data ethics review boards^30^, thereby transforming consent from a one-time transaction into an ongoing co-design process^31^. In doing so, mental health AI could evolve from its current extractive data relations to a justice-oriented framework for the governance and use of patient data^32^.

## Conclusion

Today, the patients who can most benefit from additional mental health support are also the least likely to read or understand complex terms of service—and as with HeLa cells, data may already be extracted from communities that will be the last to access the innovations their data helped create. Although separate and explicit opt-in consent may create barriers for some users, and patient-led governance may slow innovation or face industry opposition, the digital mental health sector risks repeating a shameful chapter of progress built on exploitation without such policies. The challenge, therefore, is not to halt innovation or abandon informed consent, but to ensure that progress advances with both individual choice and accountability to the communities most affected. Protecting autonomy and confidentiality must remain a foundation for ethical innovation, not an externality to be managed after the fact.

## Data Availability

No datasets were generated or analysed during the current study.
